# Global Lipidomic Analysis of Lytic KSHV Infection: The Lipid Chaperone FABP4 Supports Maximal Infectious Virion Production

**DOI:** 10.3390/v18080875

**Published:** 2026-08-11

**Authors:** Eranda Berisha, Erica L. Sanchez

**Affiliations:** Department of Biological Sciences, The University of Texas at Dallas, Richardson, TX 75080, USA; eranda.berisha@utdallas.edu

**Keywords:** Kaposi’s Sarcoma Herpesvirus (KSHV), Kaposi’s Sarcoma (KS), fatty acid binding proteins (FABPs), fatty acid binding protein 4 (FABP4), triacylglycerols (TGs), cholesterol esters (CEs), phosphatidylcholine (PC), phosphatidylethanolamine (PE)

## Abstract

Kaposi’s Sarcoma Herpesvirus (KSHV), an enveloped double-stranded DNA virus, is the etiological agent of Kaposi’s Sarcoma (KS), an endothelial cell-based tumor. KSHV is a leading cause of infection-related cancers in sub-Saharan Africa and immunocompromised individuals worldwide. Therefore, it is vital to identify the underlying mechanisms of viral infection and transmission to effectively identify specific therapeutic strategies and combat the disease. Here, we demonstrate that KSHV rewires the host cell lipidome during lytic infection. Bulk lipidomic analysis shows significant changes in the abundance of neutral lipids and phospholipids during lytic infection. We further investigated fatty acid binding proteins (FABPs) to understand the underlying mechanisms that support KSHV pathogenesis. Using the doxycycline-inducible iSLK.BAC16 cell line, we find that FABP genes are differentially regulated by lytic KSHV infection compared to latent infection. We report that FABP4 is significantly upregulated during lytic infection. FABP4 knockdown using siRNA or inhibition of the FABP4 protein via treatment with a competitive inhibitor during lytic infection significantly reduces extracellular viral titers, indicating that FABP4 supports maximal infectious virion production. This study highlights the role of FABP4 as a host target that facilitates KSHV infection and pathogenesis.

## 1. Introduction

Kaposi’s Sarcoma (KS) is an endothelial cell-based tumor endemic to sub-Saharan Africa and the Mediterranean, and the most common cancer in HIV-infected individuals [1,2,3]. Kaposi’s Sarcoma-Associated Herpesvirus (KSHV), the causative agent of KS, is an oncogenic enveloped double-stranded DNA virus and member of the Gammaherpesvirinae subfamily. KSHV infection induces the expression of several genes and metabolic changes in the host that facilitate maximal virion production. KSHV has both latent and lytic viral phases, with ~95% of KS tumor cells undergoing latent infection and only 1–5% of cells lytically replicating [1,2,3,4]. The latent phase of KSHV is marked by limited viral gene expression, no virion production, and host cell survival. However, upon reactivation, all viral genes are expressed, and virion production occurs, eventually leading to host cell lysis [4,5]. Ultimately, both the latent and lytic phases are essential for KSHV pathogenesis.

Previous publications have reported significant alterations in host cell metabolism, specifically lipid metabolism, that occur during the latent phase of KSHV infection in endothelial cells [6,7,8,9]. Latent KSHV infection induces de novo lipogenesis and requires fatty acid synthesis for survival. Furthermore, treatment with fatty acid synthesis inhibitors induces apoptosis of latently infected cells [7]. An interplay between increased lipogenesis and lipid droplet formation, cytoplasmic organelles that store neutral lipids, occurs during latent infection [6,7,8]. Another study has reported that latent infection induces an increase in cholesterol ester (CE) synthesis and CE content in lipid droplets for membrane biogenesis [9]. However, lipid droplet degradation generates free fatty acids that can be used to produce ATP through mitochondrial ꞵ-oxidation or be utilized for peroxisomal ꞵ-oxidation [6,8,9]. Interestingly, there are limited reports of lipidomic alterations during KSHV lytic infection.

The lytic cascade and the distinct stages of lytic KSHV infection are triggered by the activation of the expression of the replication and transcription activator (RTA). RTA-induced reactivation from latency is followed by immediate-early (IE) and early (E) gene expression, viral genome replication, late (L) gene expression, and finally virion assembly and egress [10]. In a previous study, lytic KSHV endothelial cells treated with 5-tetradecyloxy-2-furoic acid (TOFA), a known allosteric inhibitor of acetyl-CoA carboxylase-α (ACCA) or fatty acid synthesis, showed unaltered viral gene expression and replication. However, both intra- and extracellular infectious virion production was heavily reduced. Transmission electron microscopy (TEM) showed that TOFA-treated cells resulted in oddly shaped virion capsids while nuclear virus factories remained unaffected. It was concluded that inhibition of fatty acid synthesis alters KSHV assembly and egress [11]. Similarly, in murine herpesvirus 68 (MHV-68), a homologous gammaherpesvirus that infects mice, inhibition of lipogenesis by the drug TOFA, and subsequent viral titers during lytic infection, showed that virion production was significantly reduced. Lipidomic analysis of lytic MHV-68-infected cells revealed that lipid remodeling occurs during the early and late phases of infection, resulting in early lipogenesis and triacylglycerol (TG) uptake and subsequent lipolysis, lipogenesis, or both. There was also a gradual decrease in CEs observed [12]. In another study, in lytic KSHV-infected endothelial cells, 3 to 24 days postinfection, TG content in lipid droplets increased to support virion assembly [6,9]. Another study reported that the attenuation of the cholesterol synthesis pathway in macrophages, via treatment with statins, decreased in vitro MHV-68 replication [13]. Another group has reported that cholesterol depletion in iSLK.BAC16 cells disrupts the integrity of lipid rafts, intracellular signaling and transport micro-domains, which results in significantly reduced virion egress 4.5 days after lytic KSHV induction [14]. These studies reveal that alterations to cholesterol homeostasis occur during the lytic phase of gammaherpesvirus infection. This partly indicates an altered host lipidome, and therefore, it is essential to examine and validate all alterations to the lipidome during lytic KSHV infection as well. Although some alterations to lipid metabolism during the lytic phase of KSHV infection are reported, the entire lipidome and the molecular mechanisms that drive such alterations remain unknown.

Fatty acid binding proteins (FABPs) are cytosolic proteins that play a central role as lipid chaperones. They traffic long-chain fatty acids to specific cellular compartments for the purpose of modulating lipid metabolism through oxidation, enzyme activity regulation in the cytosol, storage, lipid-mediated transcriptional regulation in the nucleus, paracrine/autocrine signaling outside the cell, and membrane synthesis and signaling in the ER [15,16,17]. There are twelve known FABPs, ten of which are mammalian (FABP1-9, 12), named and classified based on the tissues they are most abundantly expressed in. Despite their classification, FABPs are not tissue- or cell-specific [18]. A previous study showed that in normoxic and hypoxic KSHV-infected BJAB (a human B cell line) cells, FABP1, FABP4, and FABP7 were significantly upregulated [19]. Similarly, in our KSHV-infected iSLK cell line (iSLK.BAC16), FABP4 and FABP7 were observed to be significantly upregulated during the lytic phase of infection. A study published on Severe Acute Respiratory Syndrome Coronavirus 2 (SARS-CoV-2), an RNA virus, established that FABP4 colocalizes with replication organelles, and inhibition of FABP4 impairs virus replication [20]. In breast cancer, FABP4 and CD36 (fatty acid importer) directly interact for the purpose of regulating lipid metabolism and fatty acid transport into the cell [21].

In this study, we demonstrate that KSHV rewires the host lipidome during lytic KSHV infection and that FABP4 plays a role in KSHV virus production. We report that early and late lytic gene expression is significantly reduced upon FABP4 knockdown; however, FABP4 drug inhibition significantly impacts only late lytic gene transcription [22]. Loss of and inhibition of FABP4 protein during lytic KSHV infection culminates in a significant reduction in extracellular virus titers, indicating that lytically induced FABP4 upregulation supports maximal infectious virion production. Overall, we report the significance of FABP4 during lytic KSHV infection. Through this study, we have uncovered lipid-related metabolic alterations and novel targets of lytic infection, identifying FABP4 as a potential biomarker of KSHV lytic infection.

## 2. Materials and Methods

### 2.1. Cells and Media

The cell lines used in this study are as follows: inducible SLK cells containing KSHV BAC16 (iSLK.BAC16) and inducible SLK cells (iSLK) used as a control. The iSLK.BAC16 cell line stably maintains the KSHV genome. It is latently infected with recombinant KSHV BAC16, which encodes constitutive expression of GFP. These cells also encode the viral replication and transcription activator (RTA) transgene, which is doxycycline (Dox) and sodium butyrate (NaB) inducible. This triggers the transition in iSLK.BAC16 from the latent to lytic phase of infection [23,24,25]. iSLK cells do not contain the KSHV genome and therefore are used as a control in our experiments [25]. iSLK.BAC16 cells were cultured in DMEM media (Gibco, 1195065) supplemented with 10% FBS (Corning, 30-249-CR), 10 mm L-Glutamine (Gibco, 25030081), 1 µg/mL puromycin (Mirus, 22103916), 250 µg/mL G-418 (Fisher Bioreagents, BP673-5) and 1000 µg/mL hygromycin (Corning, 30-240-CR) at 37 °C and 5% CO_2_. iSLK cells were cultured in similar media and conditions, but without hygromycin.

### 2.2. siRNA Knockdown

iSLK and iSLK.BAC16 cells were seeded at 1 × 10^5^ cells/well onto a 6-well plate. At 24 h post-seeding, cells were transfected with 25 pmol FABP4 siRNA (siFABP4, Qiagen GeneGlobe ID: S100026033) (target sequence: CAATATGGTTACGTTGGTTAA) or AllStars Negative Control siRNA (siASN, Qiagen #1027295) using Lipofectamine^®^ RNAiMAX (Invitrogen #13778-150) in Opti-MEM^®^ Medium (Gibco #11058-021) following the manufacturer’s instructions. siRNA-transfection of iSLK and iSLK.BAC16 cells occurred at 37 °C and 5% CO_2_ atmosphere for 24 h. Next, cells were treated with or without Dox and NaB (Dox/NaB) for 48 h. Dox/NaB treatment is required to induce lytic reactivation in iSLK.BAC16 cells.

### 2.3. Drug Inhibition

iSLK.BAC16 cells were seeded at 1 × 10^5^ cells/well onto a 6-well plate. At 24 h post-seeding, cells were treated with or without 50 µm BMS-309403 (MCE #HY-101903) and Dox/NaB [22]. FABP4 inhibition and lytic reactivation of iSLK.BAC16 cells occurred at 37 °C and 5% CO_2_ atmosphere for 48 h. Dox/NaB treatment is required to induce lytic reactivation in iSLK.BAC16 cells.

### 2.4. Cell Viability

iSLK.BAC16 cells were seeded at 2 × 10^4^ cells/well onto a 12-well plate. At 24 h post-seeding, cells were treated with or without 2 µm, 10 µm, 25 µm, 50 µm, 60 µm, 75 µm, 100 µm BMS-309403 (MCE #HY-101903), and 10% DMSO. FABP4 inhibition of iSLK.BAC16 cells occurred at 37 °C and 5% CO_2_ atmosphere for 72 h. Next, manual cell counting was performed via hemocytometry. Relative cell viability was calculated by dividing the number of live cells from each treatment group by the number of live cells from the control (untreated) group. The control group was defined as 100% viable, and treatment groups are expressed as a percentage of the control. All conditions were performed in 3 biological replicates.

### 2.5. Quantitative Reverse Transcription-PCR

iSLK and iSLK.BAC16 cells were seeded at 1 × 10^5^ cells/well onto a 6-well plate. The following day, cells were transfected with either siFABP4 or siASN for 24 h. Next, cells were treated with 1 μg/mL of doxycycline (Dox) and 1 mm of sodium butyrate (NaB) for 36 h or 48 h. Alternatively, cells were treated without or with 50 uM BMS-309403 and with Dox/NaB. Next, RNA was extracted using the PureLinkTM RNA Mini Kit. Two-step quantitative real-time reverse transcription-PCR (Applied Biosystems) was used to measure the relative mRNA expression of FABP1-7, RTA, ORF45, ORF70, ORF9, ORF59, ORF26, and K8.1 using the primers listed in Table 1 in iSLK cells (Dox/NaB −/+) and iSLK.BAC16 cells (Dox/NaB −/+).

### 2.6. Western Blots

FABP4 knockdown and 48 h Dox and NaB reactivated iSLK.BAC16 cells were lysed in RIPA buffer, and total protein levels for lysates were quantified using the Pierce BCA Protein Assay Kit (Thermo Fisher Scientific, Waltham, WA, USA #23225) following the manufacturer’s specifications. For each condition, 30 µg of total protein lysate was loaded onto 4–20% 10-well Mini-PROTEAN TGX Gel (BIORAD, Hercules, CA, USA #4561094) and transferred onto PVDF membranes (BIORAD, #1620264). The membranes were blocked for 1 h on a rocker with 5% skim milk in a 0.1% TBST solution (1× Tris-buffered saline with 0.1% Tween 20) (Thermo Scientific #28360) and probed with primary antibodies diluted in 5% skim milk–TBST by rocking at 4 °C overnight. Secondary antibodies were diluted in 5% skim milk–TBST and reacted on a rocker at room temperature for 2 hr. Immunolabeled proteins were visualized using Clarity Western ECL substrate and an Amersham ImageQuant™ 800 Western blot imaging system (Cytiva). Immunodetection was performed using mouse antibodies against ORF45 (Sigma Aldrich, St. Louis, MO, USA #SAB5300153, 1:1000), K8.1 (Millipore, #MABF2678, 1:500), and rabbit antibodies against alpha tubulin (Proteintech #11224-1-AP. 1:2000). Secondary antibodies included HRP conjugated goat anti-rabbit (Jackson Immuno Research Laboratory, #111-035-003, 1:20,000) or anti-mouse IgG (Jackson Immuno Research Laboratory, #115-035-003, 1:20,000). Western blots were performed in three biological replicates, and representative Western blot images are included.

### 2.7. Extracellular Virus Titer Assay

iSLK.BAC16 cells were seeded at 1 × 10^5^ cells/well onto a 6-well plate. The following day, these cells experienced either siRNA-mediated knockdown or BMS-309403 drug inhibition. Regarding siRNA-mediated knockdown, iSLK.BAC16 experienced 24 h of siASN transfection or FABP4 knockdown. Next, after 48 h of Dox/NaB treatment, cell-free supernatants containing extracellular infectious virus were collected. Supernatant titers were introduced onto iSLK cells in a 6-well plate with a confluency of 100,000 cells per well. Regarding FABP4 drug inhibition, iSLK.BAC16 experienced 48 h of 50 µm BMS-309403 and Dox/NaB treatment. Next, cell-free supernatants containing extracellular infectious virus were collected. Supernatant titers were introduced onto iSLK cells in a 6-well plate with a confluency of 50,000 cells per well. Supernatants were then centrifuged at 2000 rpm for 10 min to remove dead cells and debris. To each well, 1 μg/mL of polybrene (EMD Millipore #TR-1003-G) was added. Next, these iSLK cells were centrifuged at 300× *g* for 1 h at 37 °C and then incubated for 5 h at 37 °C to encourage infection. The cells were then washed with PBS, and fresh media was introduced. To establish latency, cells were further incubated for 48 h at 37 °C and 5% CO_2_.

### 2.8. Flow Cytometry

Flow cytometry was used to determine the infection rate of KSHV virions based on GFP+ cells. Next, 48 h after extracellular and intracellular virus titer infection experiments, cells were washed with PBS, collected, and centrifuged at 300× *g* for 5 min. Cell pellets were then resuspended in 100 µL FACS buffer (2% FBS in PBS) and fixed with 4% PFA for 15 min incubation on ice. Cells were then washed with PBS and centrifuged at 300× *g* for 5 min at 4 °C. Cell pellets were then resuspended in 500 µL FACS buffer. A BD Fortessa SORP flow cytometer, equipped with a FITC detection filter, was used to perform flow cytometry. A record of 10,000 cellular events per sample was used during FACS data collection to achieve statistical significance. Data were analyzed by FlowJo V.10.9, in which forward scatter (FSC) and side scatter (SSC) were used to identify relevant cell populations. GFP- and GFP+ were differentiated by quadrant gating, corresponding to FITC- and FITC+ cells, respectively. Statistical significance and bar graph generation were achieved using ANOVA in GraphPad Prism 10.5.0 software.

### 2.9. Bulk Lipidomics

In 10 cm dishes, iSLK BAC16 cells were seeded and then treated with Dox (1 µg/mL) and NaB (1 mm) for 48 h to induce lytic infection. After reactivation, the cells were then washed with PBS, trypsinized, and collected in microcentrifuge tubes. The cell pellets were washed again with sodium bicarbonate and then flash frozen in liquid nitrogen. For each of the two conditions, iSLK.BAC16 cells treated with or without Dox/NaB (Dox/NaB −/+) for 48 h, 5 biological replicates were generated. The cell pellets were prepared in a single batch (*n* = 10). The samples were transported to the Northwest Metabolomics Research Center (NW-MRC) for further processing. Sample prep at NW-MRC was performed as previously described [26]. Lipid species were measured using a Sciex Lipidyzer system as described elsewhere [26,27]. The Sciex Lipidyzer mass spectrometry platform consisted of Shimadzu Nexera X2 LC-30AD pumps, a Shimadzu Nexera X2 SIL-30AC autosampler kept at 10 degrees Celsius, and a Sciex QTRAP 5500 mass spectrometer equipped with an ESI source coupled with SelexION for differential mobility spectrometry (DMS). The chemical modifier used for DMS was 1-propanol. Samples were introduced to the mass spectrometer by flow injection analysis at 8 uL/min. Each sample was injected twice, first with the DMS ON (PC, PE, LPC, LPE, PG, PI, PS and SM), and then with the DMS OFF (CE, CER d18:0, CER d18:1, DAG, FFA, HexCER, LacCER, LPG, LPI, LPS, PA and TAG). A total of 1561 lipid species (plus 69 spiked isotope-labeled lipid standards) across 20 lipid classes/sub-classes were targeted using multiple reaction monitoring (MRM) and positive/negative polarity switching electro-spray ionization. SM, DAG, CE, CER d18:0, CER d18:1, HexCER, LacCER, LPG, LPI, LPS, PA, LPE, LPC and TAG were ionized in positive and PE, PG, PI, PS, PC, and FFA in negative ionization mode. Data were acquired using Analyst 1.6.3 and processed with an open-source Shotgun Lipidomics Assistant v4.1.2 software [26]. Up to 1183 lipid species were measured across the study. For quality control, a pooled human serum sample (in-house quality control) was run at the beginning and at the end of the batch, respectively. The median CV was 9.7%, and 65% of measured lipid species had a CV value of under 15%. The final data contained absolute concentrations of each lipid species based on the known concentrations of the spiked isotopically labeled internal standard lipids. Lipidomic data were normalized by protein concentration as an internal control. In the web-based platform Metaboanalyst 6.0, lipidomic data were again normalized by log (2) transformation and auto-scaled for further analysis. Statistical analysis and graph making were performed on Metaboanalyst 6.0 and the software GraphPad Prism 10.5.0.

### 2.10. Statistical Analysis

Figures and statistical analyses were generated using GraphPad Prism 10.5.0 software. Experiments were performed at least in triplicate. Data are presented as the mean ± SEM. Experimental conditions were compared to control conditions using ANOVA or an unpaired *t*-test. Significant *p*-values are noted in each figure (* *p* ≤ 0.05, ** *p* ≤ 0.01, *** *p* ≤ 0.001, **** *p* ≤ 0.0001); ns denotes non-significant change.

## 3. Results

### 3.1. KSHV Rewires the Host Lipidome During Lytic Infection

Targeted bulk lipidomics was performed on the reporter cell line iSLK.BAC16 to generate a lipid profile of major lipid classes and species as a means of understanding alterations to the host lipid metabolism upon lytic reactivation. The iSLK.BAC16 reporter cell line is a doxycycline-inducible RTA-expressing SLK cell line that stably maintains the bacterial artificial chromosome 16 (BAC16)-KSHV [23]. iSLK.BAC16 cells were seeded and treated with Dox/NaB for 48 h to induce lytic infection. Cells were then collected and washed with sodium bicarbonate before the pellets were flash frozen. Targeted lipidomic analysis included five biological replicates for each condition, normalized to internal protein concentration.

**Figure 1:**
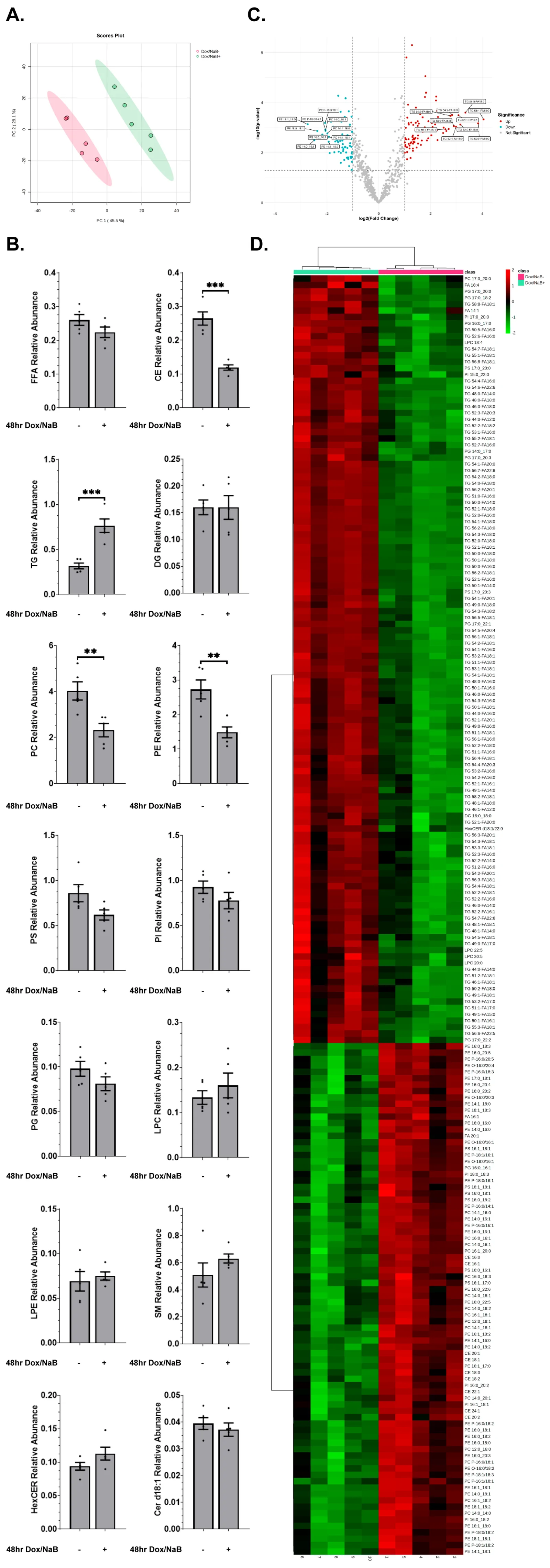
Lipidomic profiling indicates that KSHV rewires the lipidome during lytic infection. Score plots generated via the PCA classification method of Dox/NaB −/+ experimental groups show sample integrity via separation (**A**). Bar graphs show the relative abundance of major lipid classes in latent and 48 h lytically induced iSLK.BAC16 cells (**B**). The volcano plot shows the top significant lipid species in Dox/NaB+ over Dox/NaB− samples (**C**). A heatmap of the top 200 significant lipid species is displayed and generated via Metaboanalyst 6.0 software (**D**). * *p* ≤ 0.05. ** *p* ≤ 0.01. *** *p* ≤ 0.001. **** *p* ≤ 0.0001. *n* = 5.

First, we conducted principal component analysis (PCA). The 2D scores plot generated was used to visualize clustering patterns and show clear separation between the samples of each experimental condition according to its principal components [28,29,30]. The scores plot shows clear separation between our two experimental conditions (Figure 1A). Next, we determined the changes to each available lipid class, a broad category based on the lipid head group structure, upon KSHV lytic reactivation [31]. We utilized *t*-tests to statistically analyze the relative abundances (concentrations of each lipid class normalized by sample protein concentration) of each lipid class in iSLK.BAC16 cells after 48 h in the presence or absence of Dox/NaB. During lytic infection, there is a significant increase in the relative abundance of triacylglycerol (TG), while there is a significant decrease in the relative abundances of cholesterol esters (CEs), phosphatidylcholine (PC), and phosphatidylethanolamine (PE) (Figure 1B). To determine changes to each lipid species, we first normalized raw concentration values by the sample protein concentration, and then we used the free web-based software Metaboanalyst to run statistical analysis and generate both a heatmap and a volcano plot. The volcano plot displays the top significant lipid species when comparing Dox/NaB treated over non-treated conditions; the greatest significance, determined by T-tests and fold change analysis, was held by TG species (Figure 1C). The heatmap shows the top 200 significant hits, and the most significant change in Dox/NaB treated samples lies in the relative abundance of CE, TG, PC, and PE (Figure 1D). These significant changes to the lipid class and lipid species (based on chain length and bonds) in the host cell indicate that KSHV rewires the host lipidome during lytic infection [32].

### 3.2. FABP4 Is Upregulated at the Transcript Level During Lytic KSHV Infection

**Figure 2:**
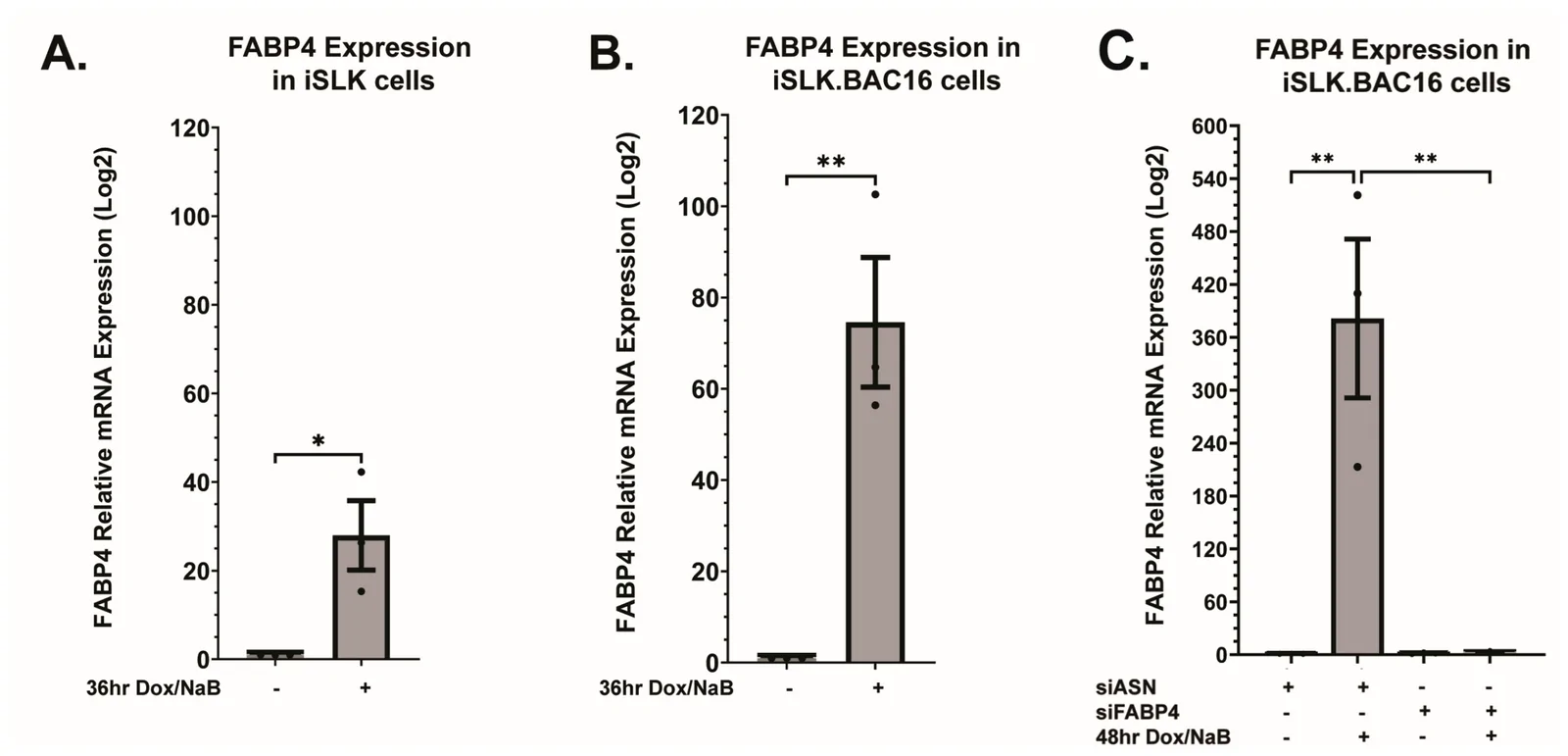
Lytic KSHV infection impacts fatty acid binding protein 4 (FABP4) at the transcript level. Fatty acid binding protein 4 (FABP4) relative mRNA expression levels indicate significant upregulation following 36 h (**A**,**B**) and 48 h (**C**) of Dox/NaB lytic reactivation in iSLK.BAC16 cells. Successful fatty acid binding protein 4 (FABP4) knockdown to basal relative mRNA expression levels was achieved in 48 h lytically induced iSLK.BAC16 cells (**C**). siASN = AllStars Negative Control siRNA. * *p* ≤ 0.05. ** *p* ≤ 0.01. *** *p* ≤ 0.001. **** *p* ≤ 0.0001. *n* = 3.

To determine the transcript levels of FABPs during lytic KSHV infection, we measured the relative mRNA expression of FABP1-7 using RT-qPCR. FABP expression was determined in both iSLK.BAC16 cells and iSLK control cells, which lack the KSHV genome, in the presence or absence of Dox/NaB [23,24,25]. Cells were transfected with either siFABP4 or AllStars Negative siRNA (siASN) as an siRNA control. At 24 h post-transfection, both iSLK.BAC16 and iSLK cells were treated for 36 or 48 h with Dox/NaB. Next, RNA was isolated, and RT-qPCR was used to measure the relative gene expression of FABP1-7. FABP4 upregulation is observed at 36 h upon Dox/NaB treatment of iSLK control cells at ~27.5-fold difference (Figure 2A). However, the upregulation of FABP4 is much higher in induced iSLK.BAC16 cells at 36 h, with an ~75-fold difference (Figure 2B). Significant FABP4 upregulation was also observed following 48 h lytic induction (Figure 2C). Although FABP5 and FABP7 were seen to be significantly expressed following the lytic reactivation of iSLK.BAC16 cells, there was a significant upregulation of FABP5 and FABP7 in our iSLK control cells treated with Dox/Nab. All other human FABPs either showed a similar significant increase upon Dox/NaB treatment in both iSLK and iSLK.BACK16 cells or were very lowly expressed in the experimental cell lines (Supplementary Figure S1A–F). Of the FABP1-7 family, FABP4 transcript levels were the most elevated during lytic KSHV infection.

To determine the role of FABP4 upregulation during lytic KSHV infection, we first used siRNA to knock down FABP4 to basal levels. Upon siFABP4 transfection, FABP4 upregulation was significantly reduced by approximately 99% in reactivated iSLK.BAC16 cells (Figure 2C). Overall, this shows that successful FABP4 knockdown was achieved via siRNA-mediated knockdown during lytic infection.

### 3.3. FABP4 Is Required for KSHV Lytic Gene Transcription

**Figure 3:**
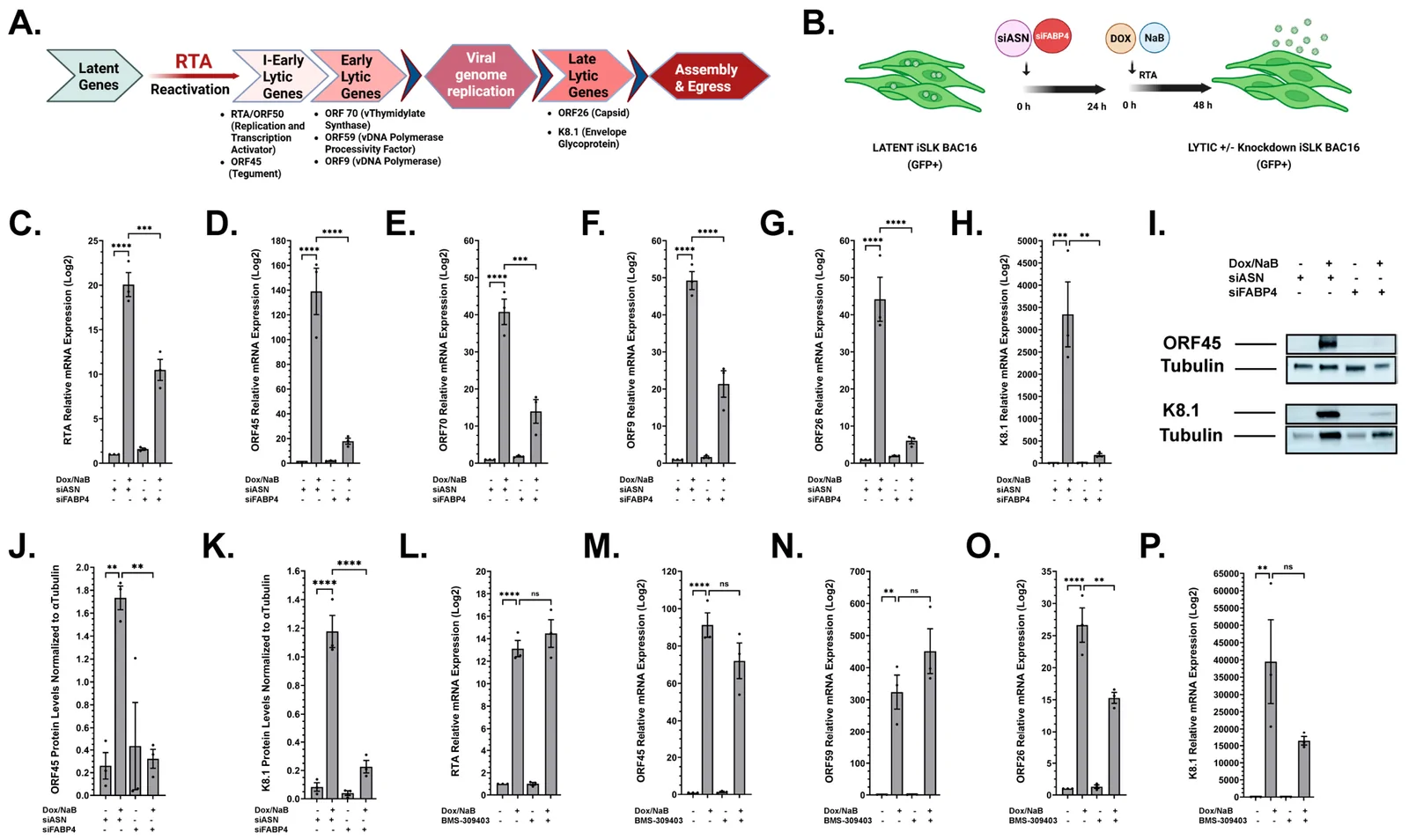
FABP4 upregulation is required for lytic gene transcription and translation. An illustrative overview of the lytic cascade, beginning with lytic induction via RTA and ending with virion assembly and egress (**A**). A schematic overview of siRNA-mediated FABP4 knockdown and 48 h of lytic reactivation of iSLK.BAC16 cells (**B**). In FABP4 knockdown and 48 h lytic-induced iSLK.BAC16 cells, the relative mRNA expression levels of immediate-early (**C**,**D**), early (**E**,**F**) and late (**G**,**H**) lytic genes are significantly reduced. Western blots show a decrease in ORF45 and K8.1 protein levels in iSLK.BAC16 cells following siFABP4 and 48 h lytic induction (**I**). Western blot quantification shows a significant decrease in ORF45 and K8.1 protein levels in iSLK.BAC16 cells following siFABP4 and 48 h lytic induction (**J**,**K**). In FABP4 (50µm BMCS-309403) inhibited and 48 h lytically induced iSLK.BAC16 cells, the relative mRNA expression levels of immediate-early (**L**,**M**), early (**N**) and late (**P**) lytic genes report no significant change. Late lytic gene ORF26 mRNA expression levels are significantly reduced following FABP4 inhibition and lytic reactivation (**O**). siASN = AllStars Negative Control siRNA. * *p* ≤ 0.05. ** *p* ≤ 0.01. *** *p* ≤ 0.001. **** *p* ≤ 0.0001. ns denotes non-significant change. *n* = 3.

We determined whether FABP4 upregulation is required for immediate-early, early and late viral gene transcription via RT-qPCR (Figure 3A). First, iSLK.BAC16 cells were transfected with siASN or siFABP4. At 24 h post-transfection, cells were induced with Dox/NaB for 48 h (Figure 3B). RNA was isolated, and RT-qPCR was performed to measure the expression of specific KSHV genes, including the immediate-early lytic genes RTA (or ORF50, the replication and transcription activator protein) and ORF45 (tegument protein); the early lytic genes ORF70 (vThymidylate synthase) and ORF9 (vDNA polymerase); and the late lytic genes ORF26 (capsid protein) and K8.1 (envelope glycoprotein [33,34,35,36,37]. Following lytic reactivation, FABP4 knockdown significantly reduced the relative transcriptional expression of RTA, ORF45, ORF70, ORF9, ORF26, and K8.1 compared to the siASN control (Figure 3C–H). To determine whether FABP4 upregulation is required for early and late viral protein translation, we performed Western blots in three biological replicates. Upon reactivation after siASN or siFABP4 treatment, iSLK.BAC16 cells were lysed in RIPA buffer to extract protein. Total cell lysates were quantified via BCA assay. Western blots revealed a decrease in the protein expression of ORF45, an early lytic gene encoding a KSHV tegument protein, upon FABP4 knockdown in lytic iSLK.BAC16 cells [35]. Additionally, the protein level of K8.1, a late lytic gene encoding a KSHV envelope glycoprotein, decreased in lytic siFABP4 samples (Figure 3I) [36]. Western blot analysis and quantification via ImageJ software (1.54g) show a significant decrease in early and late viral protein levels following FABP4 knockdown (Figure 3J,K).

To determine whether pharmacological FABP4 inhibition impacts viral gene transcription, we utilized the drug BMS-309403. First, we selected the optimal working concentration of the inhibitor, a 50µm concentration which caused no significant reduction in cell viability compared to the control (Supplementary Figure S2). Cell viability was determined by live cell counting using a hemocytometer. Absolute cell count for each treatment condition was normalized to the untreated control, which was defined as 100% viable. Next, iSLK.BAC16 cells were simultaneously treated with 50µm BMS-309403 and Dox/NaB for 48 h. RNA was isolated, and RT-qPCR was performed to measure the expression of specific KSHV genes, including the immediate-early lytic genes RTA (or ORF50) and ORF45; the early lytic gene ORF59 (vDNA polymerase processivity factor); and the late lytic genes ORF26 and K8.1 [33,34,35,36,37]. BMS-309503-mediated FABP4 inhibition (48 h) did not significantly alter the relative transcriptional expression of RTA, ORF45, ORF59, and K8.1 in lytic iSLK.BAC16 cells (Figure 3L–N,P). However, the relative expression levels of ORF26 were significantly reduced following FABP4 inhibition during lytic infection (Figure 3O). Here, we show that FABP4 plays a role in the early and late phases of lytic gene transcription.

### 3.4. FABP4 Upregulation Supports Maximal Infectious Virion Production

**Figure 4:**
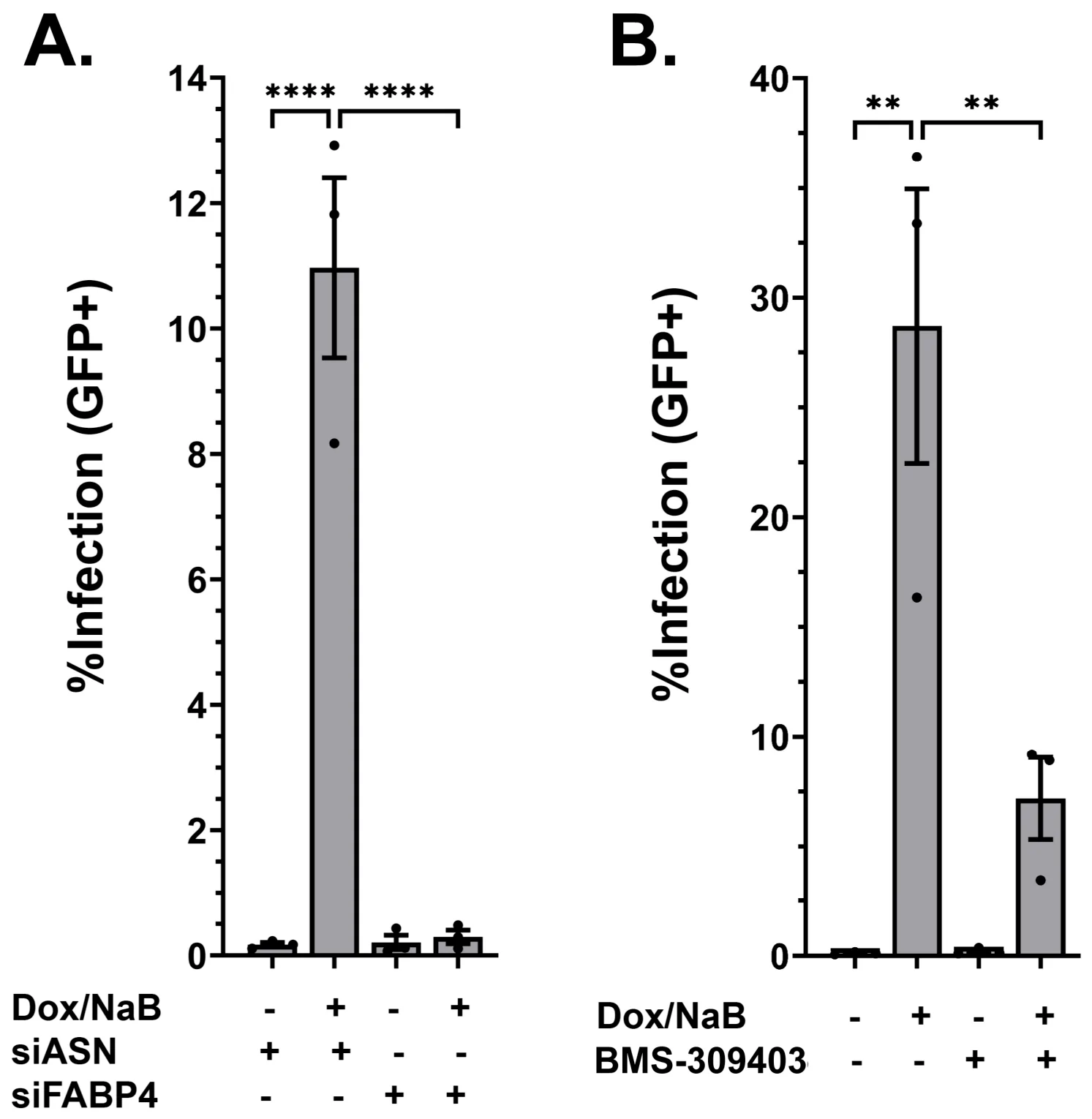
FABP4 supports maximal infectious virion production during lytic KSHV infection. Relative fluorescence intensity of infected cells to control, quantified via flow cytometry, shows a reduction in extracellular titer infection following siFABP4. (**A**) Relative fluorescence intensity of % infected cells, quantified via flow cytometry, shows a reduction in extracellular titer infection following siFABP4. (**B**) Relative fluorescence intensity of % infected cells, quantified via flow cytometry, shows a reduction in extracellular titer infection following FABP4 inhibitor BMS-309403. siASN = AllStars Negative Control siRNA. * *p* ≤ 0.05. ** *p* ≤ 0.01. *** *p* ≤ 0.001. **** *p* ≤ 0.0001. *n* = 3.

To determine whether lytically induced FABP4 upregulation supports maximal infectious virion production, we performed extracellular virus titer infection assays in triplicate. At 24 h post-siASN or FABP4 knockdown, iSLK.BAC16 cells were treated without or with Dox/NaB to induce lytic replication. At 48 h post-induction, cell-free supernatants were collected and titered onto naive iSLK control cells (100,000 cells) in the presence of 1 µg/mL of polybrene. iSLK cells were then spinoculated for 1 h and then incubated in a 37 °C incubator for 4 h. After incubation, the supernatant was removed, fresh media was added, and cells were further incubated for 48 h. Cells were then collected and prepared for flow cytometry analysis. We performed flow cytometry to measure the titer of infection or the number of virus-infected cells based on the mean fluorescent intensity for GFP+ cells using the FITC channel. The representative flow-cytometry dot plots show that upon FABP4 knockdown, significantly fewer infected cells are detected in the titer sample (GFP-expressing cells) when compared to the siASN transfection control titer sample (Supplementary Figure S3A). Upon lytic reactivation, infection with supernatant from siASN-transfected samples resulted in ~11% GFP+ cells. Meanwhile, infection with supernatant from reactivated and siFABP4-transfected samples resulted in only ~0.3% GFP+ cells (Figure 4A). These data indicate that knocking down lytically induced FABP4 upregulation significantly reduces extracellular infectious KSHV virus in iSLK.BAC16 cells.

To determine whether FABP4 pharmacological inhibition during lytic KSHV infection reduces maximal infectious virion production, we performed another extracellular virus titer infection assay. After 48 h of induced lytic reactivation and BMS-309403 treatment, cell-free extracellular virus-containing supernatants were titered onto naive iSLK cells (50,000) in the presence of 1 µg/mL of polybrene. Cells were then spinoculated for one hour and incubated in a 37 °C incubator for 4 h. After incubation, the supernatant was removed, fresh media was added, and cells were further incubated for 48 h before being trypsinized and prepared for flow cytometry analysis. The representative flow-cytometry dot plots show that significantly fewer infected cells are detected in the lytically reactivated BMS-309403-treated titer sample compared to the lytically reactivated BMS-309403 non-treated titer sample (Supplementary Figure S3B).

**Figure 5:**
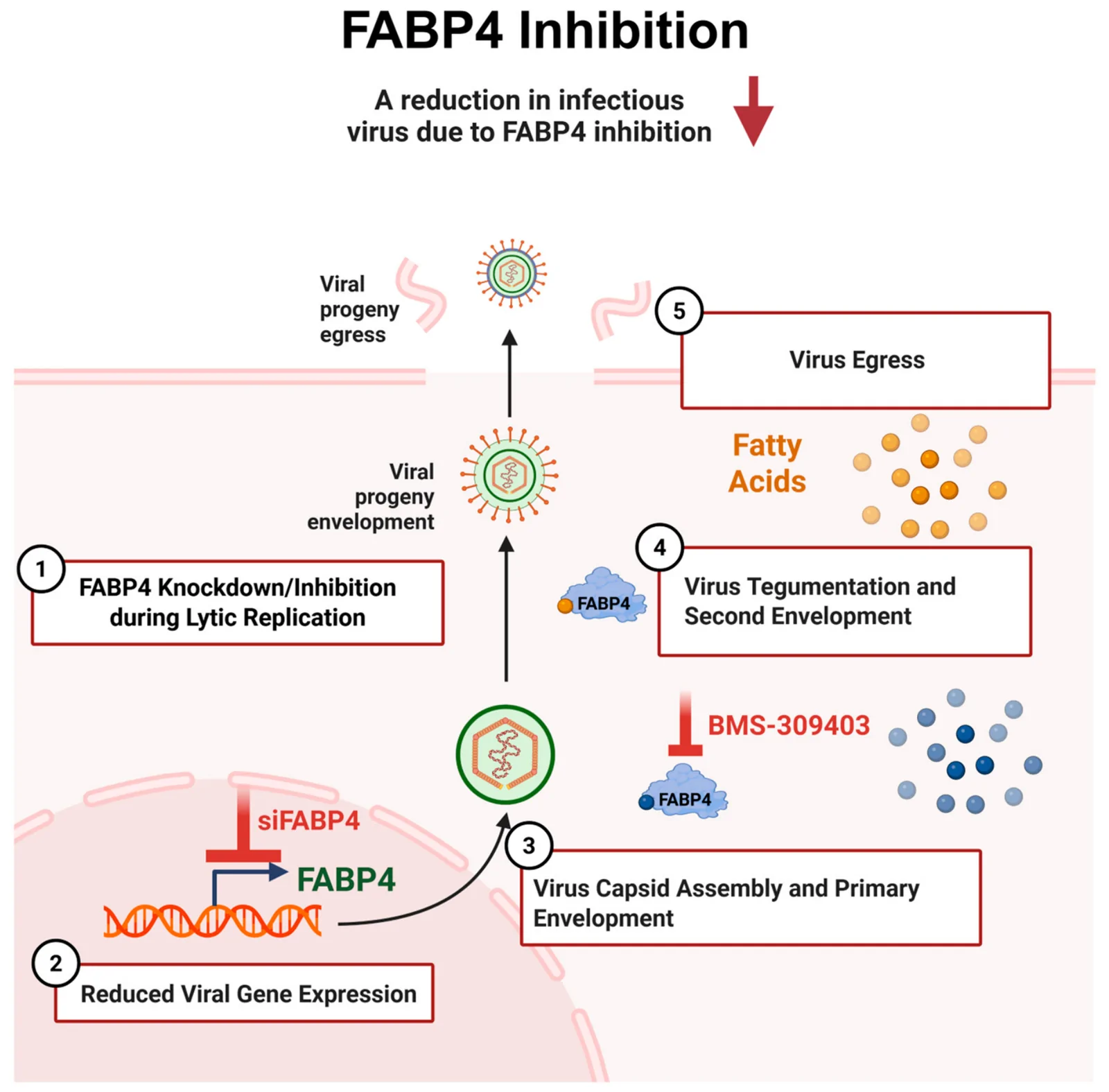
Summary model of FABP4 inhibition during lytic KSHV infection. During KSHV lytic replication, inhibition or knockdown of FABP4, either by siRNA-mediated silencing (siFABP4) or pharmacological inhibition, reduces viral gene expression and impairs the production of infectious virions. Overall, these findings identify FABP4 as a host factor that promotes efficient KSHV lytic replication and infectious virus production. Created in BioRender. Lab, S. (2026), https://BioRender.com/3ejp1xs.

Upon lytic reactivation, infection with the supernatant from BMS-309403 non-treated samples resulted in ~28.7% GFP-positive cells. Meanwhile, infection with supernatant from reactivated and BMS-309403-treated samples resulted in only ~7.2% GFP+ cells (Figure 4B). These data indicate that FABP4 inhibition significantly reduces extracellular infectious KSHV virus upon reactivation of iSLK.BAC16 cells. These data suggest that FABP4 upregulation is contributing to maximal infectious virion production during lytic KSHV infection (Figure 5). Overall, loss and chemical inhibition of FABP4 leads to the reduction of viral transcript availability, which is a critical downstream requirement for maximal infectious virion production.

## 4. Discussion

This study determines alterations to the host cell lipidome during lytic KSHV infection and highlights the underlying mechanisms that support it. Previous metabolomic studies in KSHV latently infected endothelial cells have shown a significant increase in lipid abundance compared to mock-infected samples. Furthermore, follow-up functional studies showed that lipogenesis is required for the survival of latently infected endothelial cells [7]. In our study, we used the inducible iSLK.BAC16 system and report a significant increase in the relative abundance of TGs and a significant decrease in the relative abundances of CE, PC, and PE in lytic KSHV samples compared to latent samples at 48 h post-reactivation (Figure 1B,C). It has been previously reported that TG-enrichment of lipid droplets increases during the lytic phase of KSHV infection in endothelial cells. However, this previous study was reported at very different timepoints than our study, exploring lipid alterations from three to fourteen days after lytic infection [9]. Interestingly, hepatitis C virus was reported to be dependent on its capsid protein association with TG-rich lipid droplets for virion assembly and infectious virion production [38]. Lipidomic analysis of lytic MHV-68-infected cells revealed that an early (at 8 hpi) increase in diacylglycerol (DAG) and TG, followed by a TG decrease, free fatty acid (FFA) increase, and steady state of DAG at 12 hpi. These data implicate early lipogenesis and TG uptake, followed by lipogenesis, lipolysis, or both [12]. Our reported significant increase in the relative abundance of TG following lytic induction could be due to TG-rich lipid droplets. During latency, there is an increase in CE synthesis required for microtubule formation and their possible involvement in neo-angiogenesis [9]. Regarding CE metabolism and cholesterol homeostasis, cholesterol is esterified by enzymes Acyl-CoA: cholesterol O-acyltransferases 1 and 2 (ACAT1 and ACAT2) to produce CEs, while CEs are degraded by CE hydrolases to generate fatty acids for β-oxidation and cholesterol for membranes, lipid rafts, and viral envelopes [13,39]. Cholesterol-rich lipid rafts, intracellular signaling and transport micro-domains are required for successful KSHV virion production. Cholesterol depletion and the inhibition of cholesterol biosynthesis disrupt the integrity of lipid rafts, which results in reduced KSHV virion egress [14]. Therefore, during lytic infection, the decrease in the relative abundance of CE could signify a need for cholesterol in lipid rafts and viral envelopes. Previous lipidomic studies defined the lipid composition of some enveloped viruses, three strains of influenza A virus, and SARS-CoV-2 derived from two different cell types to include high concentrations of PE and/or PC [40,41]. The reduction of the relative abundances of PC and PE in our lipidomic data suggests that these lipid classes could be utilized to support the KSHV viral envelope.

To understand the underlying mechanisms that support infectious KSHV virion production, we focused on fatty acid binding proteins (FABPs). Previously, it was reported that in KSHV-infected B-cells (BJAB), FABP1, FABP4, and FABP7 are significantly upregulated under normoxic and hypoxic conditions [19]. Hypoxia is known to induce KSHV productive replication [42]. Following FABP7 knockdown, the KSHV genome copy number was significantly reduced. However, a successful FABP4 knockdown was not generated to investigate the role of FABP4 during KSHV infection [19]. Out of the family of FABP1-7, we report that FABP4 is significantly upregulated in iSLK.BAC16 cells following 36 h and 48 h lytic reactivation (Figure 2 and Supplementary Figure S1). While Dox/NaB treatment of iSLK control cells induced FABP4 expression, FABP4 transcript levels were most elevated following Dox/NaB treatment in iSLK.BAC16 cells, suggesting a KSHV-dependent upregulation of FABP4 (Figure 2). FABP4 transcriptional regulation has been linked to the transcription factor Peroxisome Proliferator-Activated Receptor Gamma (PPARG) [43,44,45,46,47,48].

Moreover, we investigated which stage of lytic KSHV infection requires FABP4 up- regulation. The replication and transcription activator (RTA)-induced reactivation begins the lytic cascade of KSHV infection. This is followed by immediate-early (IE) gene expression, early (E) gene expression, viral genome replication, late (L) gene expression, and virion assembly and egress (Figure 3A) [10,49]. Transcript levels of the immediate-early genes RTA and ORF45, as well as the early lytic genes ORF70 and ORF9, were significantly downregulated following FABP4 knockdown in lytically reactivated cells (Figure 3C-G) [33,34,35,36,37]. Similarly, the transcript levels of ORF26 and K8.1, two late lytic genes, also showed significant downregulation. (Figure 3H-I) [33,34,35,36,37]. This transcriptional downregulation is further supported by the significant decrease in ORF45 and K8.1 protein levels (Figure 3I-K). To evaluate whether FABP4 protein inhibition recapitulates the siFABP4 phenotype, we utilized BMS-309403 to competitively block the fatty acid binding pocket of FABP4 [22]. Interestingly, we report that the transcript levels of the immediate-early genes, RTA and ORF45, as well as the early lytic gene ORF59, were not significantly altered following FABP4 inhibition in lytically reactivated cells (Figure 3L–N) [33,34,35,36,37]. Notably, the transcript level of the late lytic gene ORF26 was significantly reduced, and the transcript level of the late lytic gene K8.1 showed a reduced trend (Figure 3O,P). Overall, we observe that FABP4 upregulation is required for lytic gene transcription. This requirement potentially stems from the established role of FABP4 in regulating fatty acid oxidation (FAO) and lipolysis, as seen in breast cancer models [21,50,51,52]. Ultimately, this suggests that KSHV could induce FABP4 upregulation to exploit fatty acid energy reserves, providing metabolic fuel necessary to drive lytic gene transcription and protein translation.

Finally, we report that a significant reduction of extracellular infectious virus was observed following both the loss of FABP4 mRNA and FABP4 protein inhibition (Figure 4). Since we also observed that FABP4 upregulation is essential for viral gene transcription, the reduction in viral protein availability could act as a bottleneck preventing subsequent steps such as viral assembly and maximal infectious virion production. Interestingly, a previous study reported that inhibition of fatty acid synthesis via treatment with the drug TOFA in lytic iSLK.BAC16 cells showed unaltered viral gene expression and genome replication. However, both intra- and extracellular infectious virus production was significantly reduced, suggesting impaired viral assembly [11]. As a cytosolic lipid chaperone, FABP4 traffics fatty acids to various cellular compartments, potentially serving multiple functions in supporting maximal infectious virion production [15,16,17,21,50,51,52,53].

In conclusion, we have determined that KSHV rewires the host lipidome during lytic KSHV infection [54]. Furthermore, lytically induced FABP4 upregulation supports maximal infectious virion production. The specific mechanistic details of FABP4-dependent viral gene transcription require further investigation. With this study, we identify lipid-related metabolic vulnerabilities and highlight FABP4 as a novel target of lytic KSHV infection. Our future studies will aim to further examine the pharmacological potential of FABP4 inhibition as a strategy to attenuate KSHV pathogenesis.

## Figures and Tables

**Table 1:** Caption.RT-qPCR Primer Sequences.

Primer Name	Organism	Oligo Sequence (5′-3′)
Tubulin Fwd	Human	5′-TCCAGATTGGCAATGCCTG-3′
Tubulin Rev	Human	5′-GGCCATCGGGCTGGAT-3′
FABP1 Fwd	Human	5′-TGCCGGAAGAGCTCATCCAG-3′
FABP1 Rev	Human	5′-TTTTGGATCACTTTGGACCC-3′
FABP2 Fwd	Human	5′-ACAGTCAAAGAATCAAGCAC-3′
FABP2 Rev	Human	5′-CCCTCAAGGCTCCAGGTCCC-3′
FABP3 Fwd	Human	5′-CTACCAGGCAGGTGGCCAGC-3′
FABP3 Rev	Human	5′-CTCCACCCCCAACTTAAAGC-3′
FABP4 Fwd	Human	5′-GAGTGGGCTTTGCCACCAGG-3′
FABP4 Rev	Human	5′-TTCCTGGCCCAGTATGAAGG-3′
FABP5 Fwd	Human	5′-GGAGTGGGAATAGCTTTGCG-3′
FABP5 Rev	Human	5′-CTGTGTTGTTTTCAAAGTGC-3′
FABP6 Fwd	Human	5′-GCGTGCACATGGGTGAGCCGG-3′
FABP6 Rev	Human	5′-TCTTCTCACTCTCCATCTCG-3′
FABP7 Fwd	Human	5′-CGTGGGCTTTGCCACTAGGC-3′
FABP7 Rev	Human	5′-CTCCGTGTTCTTGAATGTGC-3′
RTA Fwd	KSHV	5′-CGCAATGCGTTACGTTGTTG-3′
RTA Rev	KSHV	5′-GCCCGGACTGTTGAATCG-3′
ORF45 Fwd	KSHV	5′-CAACTCTCCGGACGTGAACA-3′
ORF45 Rev	KSHV	5′-GGAGATTGGGTTGGGAGGTG-3′
ORF59 Fwd	KSHV	5′-GACAGCGTCTCGCTGACAGA-3′
ORF59 Rev	KSHV	5′-CACACGCGTGAGCTATTCGG-3′
ORF70 Fwd	KSHV	5′-TCCGCCCTGTCACTTGTTAT-3′
ORF70 Rev	KSHV	5′-GCGTTTTGTAGATGTGGGCA-3′
ORF9 Fwd	KSHV	5′-CACCCCTCATAACAACCCCT-3′
ORF9 Rev	KSHV	5′-CGAGCCCCATGATGACAAAG-3′
ORF26 Fwd	KSHV	5′-AGCCGAAAGGATTCCACCATT-3′
ORF26 Rev	KSHV	5′-CCGTGTTGTCTACGTCCAGA-3′
K8.1 Fwd	KSHV	5′-TAACCATGAGTTCCACACAGATTC-3′
K8.1 Rev	KSHV	5′-GGTTTTGTGTTACACTATGTAGG-3′

## Data Availability

The original contributions presented in this study are included in the article/Supplementary Materials. Further inquiries can be directed to the corresponding author(s).

## Supplementary Materials

The following supporting information can be downloaded at: https://www.mdpi.com/article/10.3390/v18080875/s1, Figure S1: FABP regulation during lytic KSHV infection; Figure S2: Relative cell viability post FABP4 drug inhibition; Figure S3: Titer infections following FABP4 knockdown and drug inhibition; Table S1: Bulk lipidomic data (lipid class) normalized to protein concentration; Table S2: Bulk lipidomic (lipid species) data normalized to protein concentration.

## Abbreviations

The following abbreviations are used in this manuscript:

KSHV	Kaposi’s Sarcoma-Associated Herpesvirus
KS	Kaposi’s Sarcoma
RTA	Replication and Transcription Activator
ORF45	Tegument Protein
ORF9	Viral DNA Polymerase
ORF70	Viral Thymidylate Synthase
ORF59	Viral DNA Polymerase Processivity Factor
ORF26	Capsid Protein
K8.1	Viral Glycoprotein
FABP4	Fatty Acid Binding Protein 4
TG	Triacylglycerol
CE	Cholesterol Esters
PC	Phosphatidylcholine
PE	Phosphatidylethanolamine
